# Lidocaine Enhances Contractile Function of Ischemic Myocardial Regions in Mouse Model of Sustained Myocardial Ischemia

**DOI:** 10.1371/journal.pone.0154699

**Published:** 2016-05-03

**Authors:** Björn Müller-Edenborn, Gabriela Kania, Elena Osto, Philipp Jakob, Nazmi Krasniqi, Beatrice Beck-Schimmer, Przemyslaw Blyszczuk, Urs Eriksson

**Affiliations:** 1 Cardioimmunology, Center of Molecular Cardiology, University of Zurich, Wagistr. 12, CH-8952, Schlieren, Switzerland; 2 Research of Systemic Autoimmune Diseases, Division of Rheumatology, University Hospital Zurich, Wagistr. 14, CH-8952 Schlieren, Switzerland; 3 Center of Molecular Cardiology, University of Zurich, Wagistr. 12, CH-8952 Schlieren, Switzerland; 4 Laboratory of Translational Nutrition Biology, Eidgenössische Technische Hochschule Zürich, Schorenstrasse 16, 8603 Schwerzenbach, Switzerland; 5 Institute of Physiology, University of Zurich, Winterthurerstr. 190, CH-8057 Zurich, Switzerland; 6 Department of Medicine, GZO-Zurich Regional Health Center, Spitalstr. 66, CH-8620, Wetzikon, Switzerland; 7 Department of Cardiology, University Heart Center, University Hospital Zurich, Raemistr. 100, CH-8001, Zurich, Switzerland; 8 Institute of Anesthesiology, University Heart Center, University Hospital Zurich, Raemistr. 100, CH-8001, Zurich, Switzerland; Emory University, UNITED STATES

## Abstract

**Rationale:**

Perioperative myocardial ischemia is common in high-risk patients. The use of interventional revascularisation or even thrombolysis is limited in this patient subset due to exceedingly high bleeding risks. Blockade of voltage-gated sodium channels (VGSC) with lidocaine had been suggested to reduce infarct size and cardiomyocyte cell death in ischemia/reperfusion models. However, the impact of lidocaine on cardiac function during sustained ischemia still remains unclear.

**Methods:**

Sustained myocardial ischemia was induced by ligation of the left anterior descending artery in 12–16 weeks old male BALB/c mice. Subcutaneous lidocaine (30 mg/kg) was used to block VGSC. Cardiac function was quantified at baseline and at 72h by conventional and speckle-tracking based echocardiography to allow high-sensitivity *in vivo* phenotyping. Infarct size and cardiomyocyte cell death were assessed post mortem histologically and indirectly using troponin measurements.

**Results:**

Ischemia strongly impaired both, global systolic and diastolic function, which were partially rescued in lidocaine treated in mice. No differences regarding infarct size and cardiomyocyte cell death were observed. Mechanistically, and as shown with speckle-tracking analysis, lidocaine specifically improves residual contractility in the ischemic but not in the remote, non-ischemic myocardium.

**Conclusion:**

VGSC blockade with lidocaine rescues function of ischemic myocardium as a potential bridging to revascularisation in the setting of perioperative myocardial ischemia.

## Introduction

Outcome of acute myocardial ischemia remarkably improved with the availability of interventional and pharmacological revascularisation techniques. Unanticipated myocardial ischemia in the perioperative phase however is frequent in high-risk patients and substantially affects morbidity and mortality [1]. Unfortunately, percutaneous coronary intervention with subsequent need for dual anti-platelet therapy or pharmacological thrombolysis carries an exceedingly high bleeding risk in this patient subset.

Myocardial ischemia leads to accumulation of intracellular sodium (Na^+^_i_) and, through mechanisms including the sacrolemmal Na^+^/Ca^++^ exchanger and other pathways, an increase in intracellular calcium (Ca^++^_i_)_._ This accumulation of Ca^++^_i_ during ischemia is deleterious as it results in increased diastolic wall tension, myocardial contractile work and oxygen consumption [2,3]. The compression of the vascular space during diastole reduces remaining coronary and myocardial blood flow, resulting in a viscious circle of increased myocardial oxygen demand and decreased oxygen supply.

Na^+^ can enter the cell through voltage-gated sodium channels (VGSC). This not only occurs when VGSC are opened during the upstroke of the action potential, but also through persistent Na^+^ current. Under normal conditions, persistent Na^+^ current represents a small fraction of the peak gradient only. However, it is greatly enhanced under hypoxic conditions and is thought to largely contribute to the increase in Na^+^_i_ during ischemia [4–6].

Previous studies have demonstrated that lidocaine, a local anesthetic and class Ib antiarrhythmic that blocks VGSC, reduces Na^+^_i_ and prevents Ca^++^-overload in myocardial ischemia [7]. Conversely, application of lidocaine during myocardial ischemia was demonstrated to improve functional parameters such as cardiac contractility in highly artificial experimental settings of explanted and perfused Langendorff-hearts, or of static parameters such as histological infarct size in very short term *in vivo* models[7–9]. Moreover, data derived from *in vitro* studies on isolated cardiomyocytes suggest a possible impact of VGSC blockade on diastolic cardiac function[10]. However, the relevance of these observations in the more complex pathophysiological response that occurs during, and within the first days after myocardial ischemia remains unclear.

We hypothesized that administration of lidocaine might lessen the deterioration of cardiac function in sustained myocardial ischemia, allowing to bridge the time until revascularisation can be performed with a reasonable risk for bleedings. To keep the influence on cardiovascular function as small as possible, we used *in vivo* phenotyping with high-perfomance echocardiography techniques requiring only minimal anesthesia.

## Methods

### Experimental Myocardial Infarction

All animal procedures were approved by the institutional Animal Care and Use Committee of the University of Zurich, Switzerland and were in accordance with swiss federal law. Twelve to sixteen weeks old and weight matched BALB/c mice were obtained from the local breeding facility and housed in a temperature-controlled environment on an alternating 12h light/dark cycle.

Myocardial infarction was induced by ligation of the left anterior descending artery (LAD) as described before[11]. Briefly, mice were intubated with a 20 gauge polypropylene tube after induction of anesthesia (4% isoflurane) and ventilated with a small animal volume-control ventilator (Harvard Apparatus, Holliston, MA) at tidal volumes of 280 μl and 120 cycles per minute. Anesthesia was maintained with 2% of isoflurane. The thoracic cavity was opened by left-sided thoracotomy and the heart exposed. The LAD was visualized and ligated with an 8–0 suture just distal of its appereance under the left atrial appendage. This induces a well reproducible and large area of ischemic myocardium that is easily distinguishable macroscopically from the non-ischemic myocardium. The thoracic cavity and skin were then closed with 4–0 suture and the animal was extubated after restoration of spontaneous respiration. Sham operated mice (n = 8) underwent the same procedure, with the exception that the 8–0 suture was not tightened. All animals received analgesia with 2 mg/kg bodyweight flunixin meglumine in 12h intervalls. Postoperatively, all animals were checked twice daily for specified signs of stress and pain (spontaneous activity, posture, mouse grimace scale and weight). Animals were euthanized using 10 minutes of insufflation with carbon dioxide when meeting pre-specified criteria or the 72h endpoint following echocardiography. A detailed outline of these criteria can be found in S1 File. Mortality in the first 72h (including mice that were euthanized according to the prespecified criteria) following LAD ligation was 35%, which is expected in this model [12–14]. An outline of the experimental protocol can be found in S1 Fig.

### Echocardiography

Echocardiography was performed using a digital small animal ultrasound system (Vevo 2100 Imaging System, VisualSonics, Toronto, Canada) with a 18 to 38 Mhz linear-array probe[15]. Animals were lightly sedated with isoflurane and placed on a heating pad. To keep the influence of anesthesia on cardiovascular physiology during echocardiography as small as possible, all mice were anesthesized with a constant of 2% of isoflurane applied via a face mask, maintaining heart rates of >400 bpm. The complete aquisition of all images required approximately 8 minutes per mouse. All examinations were performed by a single investigator (EO) blinded for the experimental groups. Standardized two-dimensional long- and short axis views as well as apical 4-chamber views were obtained from all animals to assess LV size and function. Left ventricular ejection fraction (EF) was calculated as ([end-diastolic volume—end-systolic volume]/end-diastolic volume) x 100. Left ventricular fractional area change (FAC) was calculated as ([end-diastolic area—end-systolic area)/end-diastolic area) x 100. Longitudinal strain (LS) was calculated as the change of length of a specific segment divided by its original length ([L_1_-L_0_]/L_0_).

Pulsed-wave Doppler was used in the apical 4-chamber view to measure early (E) and late (A) mitral inflow velocities. Early (Ea) and late (Aa) mitral valve velocities were quantified by tissue Doppler imaging. Due to the in our experience insufficient image quality in the apical 4-chamber views in mice following thoracotomy, tissue Doppler was performed in the parasternal short-axis view on the level of the chordae tendineae as described before[16].

Analysis of all images was performed offline by two (BME, EO) independent, blinded investigators for the conventional measurements and by one (BME) blinded investigator for the semi-automated strain measurements using the Vevo2100 Software Version 1.6. The inter-observer-variability was low with an intra-class-correlation coefficient for EF and FAC of 0.93 and 0.92, respectively.

### Randomization and Experimental Protocol

Animals were randomized to the control or treatment group prior to the baseline echocardiography using a free online randomizer (*www*.*randomizer*.*org*). Animals of the treatment group received a total of six doses of 30mg/kg bodyweight of lidocaine (lidocaine hydrochloride, 10mg/ml, Sintetica, Mendisio, Switzerland). We chose the subcutaneous route to allow for a sustained drug effect and decreased toxicity. The reported LD50 of subcutaneous lidocaine in mice is 200–400 mg/kg (summary report 1999, committee for veterinary medicinal products, EMEA). Animals were injected in 12h intervals, starting 30 minutes following LAD ligation. Animals of the control group received an equal volume of phosphate-buffered saline in the before mentioned interval.

### Troponin Measurements

Blood was harvested by puncture of the inferior vena cava. Troponin T was quantified using the Elecysy Troponin T high sensitive system (Roche, Basel, Switzerland). The detection limit of this assay is 3 pg/ml.

### Histological Quantification of Infarct Size

Hearts were fixed in 4% formaldehyde, cut into three transverse slices of equal size (basal, mid ventricular and apical level) parallel to the atrioventricular groove as described before [15] and embedded in paraffin. For Masson-Trichrome staining, slides were deparaffinized in 70% ethanol, followed by incubation steps in iron-hematoxylin, ponceau xylidine/fuchsin acid/azophloxin and phosphotungstic acid hydrate-Orange-G followed by counterstaining with waterblue, with several washing steps using acetic acid or distilled water in between.

Total LV endocardial circumference and the endocardial circumference occupied by scar tissue staining positive for Masson-Trichrome at the basal, mid ventricular and apical level was analysed in a semi-automatic fashion by a blinded investigator using imageJ (Bethesda, MD). Infarct size was then calculated as the mean percentage of endocardial circumference occupied by the scar tissue of the LV.

### Statistical Analysis

Mortality between mice of the control and treatment group was compared using the Log-rank test. Data was checked for normality using Kolmogorov-Smirnow-test. Normally distributed data were analysed using t-test (two groups) or One-way-ANOVA with Holm-Sidak posthoc test (> two groups).

Non-normally distributed data was compared using the Kruskal-Wallis-test with Dunn posthoc test (> 2 groups) or Mann-Whitney-test (2 groups).

Intraclass-correlation to compare the interobserver agreement of the two independent echo analysts was calculated for absolute agreement in two-way mixed mode. Hotelling T-Test was used in the analysis of mitral flow velocities.

A p < 0.05 was considered significant. Statistical calculations were carried out using either SPSS Statistics Version 22 (IBM, Armonk, NY) or Graphpad Prism Version 6 (Graphpad Software, La Jolla, CA).

## Results

### Preoperative Characteristics and Echocardiographic Validation of Myocardial Ischemia Model

Preoperative descriptive and baseline echocardiographic values did not differ between animals that were randomized to the treatment or control group (Table 1).

**Table 1:** Descriptive preoperative values of control and treatment mice.

	Control	lido	
	n = 8	n = 8	
body weight (g)	30,56 (0,78)	31,03 (0,53)	*n*.*s*.
heart rate (bpm)	413 (9,58)	435 (11,31)	*n*.*s*.
EF (%)	56,18 (0,38)	54,0,6 (0,99)	*n*.*s*.
FAC (%)	38,36 (0,69(	36,62 (0,76)	*n*.*s*.
EDV* (ml)	71,61 (3,32)	66,23 (2,04)	*n*.*s*.
area* (sq mm)	27,66 (0,53)	26,74 (0,81)	*n*.*s*.
LS (%)	-10,935 (0,466)	-8,94 (0,92)	*n*.*s*.

*measured at end- diastole

*n*.*s*. *non-significant*

*EF ejection fraction*, *FAC fractional area change*, *EDV end-diastolic volume*, *LS longitudinal strain*

Ligation of the LAD resulted in an average histological infarct size 72h following ligation of 45.3% of the left ventricular circumference, which is in line with reported results for this model[8,15]. Mortality within the first 72h following LAD ligation was 35%, comparable to what others report[12–14]. No difference was observed between mice of the control and lidocaine group (S2 Fig; p = 0.77).

Global systolic LV function in terms of EF, FAC as well as speckle-tracking based measurements of LS was drastically impaired with LS demonstrating the strongest percentage change (S3A and S3B Fig)[15]. Regional analysis showed that functional impairment is limited to ischemic LV segments of the infarct zone (S3C and S3D Fig). Mice developed diastolic dysfunction with a restrictive filling pattern (S4A and S4B Fig).

### Lidocaine treatment rescues global systolic and diastolic LV function

**Fig 1:**
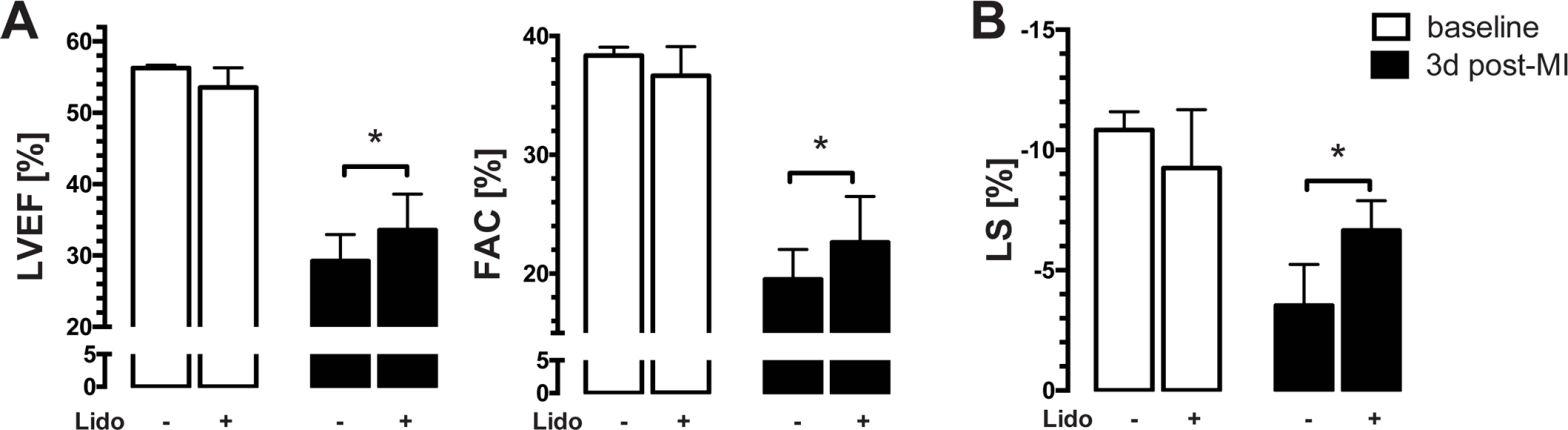
Lidocaine treatment rescues global systolic function during ischemia. Absolute values of conventional echocardiographic parameters EF and FAC **(A)** and speckle-tracking derived LS **(B)** before (white bars) and 72h following LAD ligation (black bars) with lidocaine or vehicle are given. EF, FAC and LS are partially rescued with lidocaine (*p = 0.0184 for EF; *p = 0.0343 for FAC; *p = 0.0198 for LS). *EF ejection fraction*, *FAC fractional area change*, *LS longitudinal strain*.

LAD ligation led to a deterioration of global LV systolic function. Functional impairment, however, was reduced in lidocaine-treated mice (Fig 1A, p = 0.0184 for EF, p = 0.0343 for FAC). This effect was also evident using speckle tracking analysis with improved LS in mice receiving lidocaine (Fig 1B, p = 0.0198 for LS).

**Fig 2:**
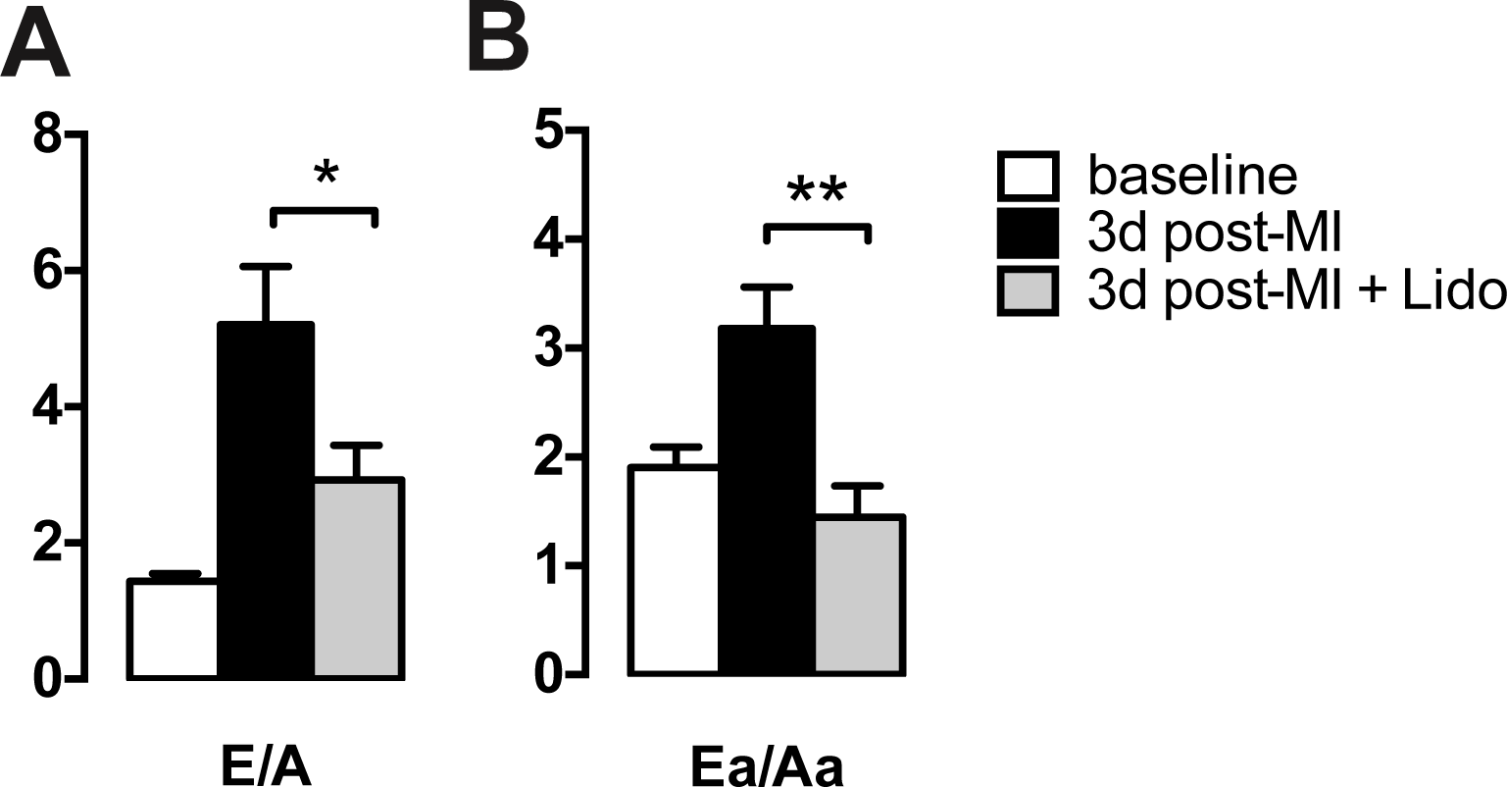
Lidocaine treatment attenuates diastolic dysfunction during ischemia. **A + B** Ratios of mitral inflow velocities (E/A) and mitral valve anulus velocities (Ea/Aa) are preserved following LAD ligation in mice receiving lidocaine (gray bars) as compared to mice receiving vehicle instead (black bars) and are comparable to preoperative values (white bars). p* = 0.0078, p**<0.0001.

The development of restrictive filling and diastolic dysfunction with LAD ligation as evidenced by a strong increase in the E/A and Ea/Aa ratios was blunted with lidocaine (Fig 2A and 2B).

### Lidocaine Treatment Does Not Affect Cardiomyocyte Necrosis and Infarct Size

**Fig 3:**
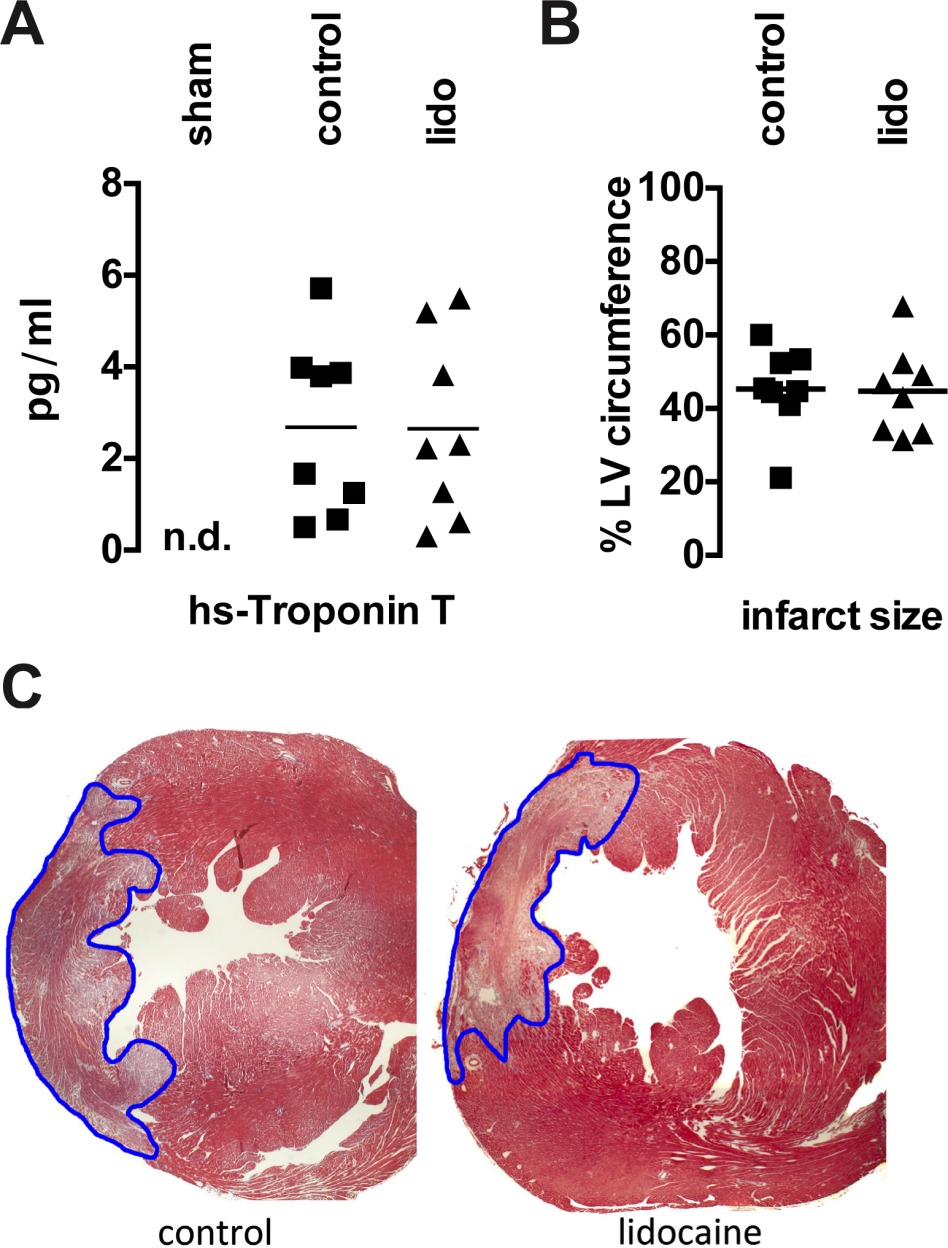
Lidocaine does not affect cardiomyocyte cell death. **A** High-sensitivity (hs)-Troponin T is equally elevated 72h following LAD ligation in mice receiving lidocaine (lido) or vehicle (control). **B** Infarct size given as percentage circumference of the left ventricle that is infarcted is comparable in mice receiving lidocaine and in control animals. **C** Representative slides from the mid-ventricular section of hearts 3 days following LAD ligation and stained with masson-trichrome. *n*.*d*. *not detectable; LV left ventricle*.

Serum troponin as a marker of cardiomyocyte necrosis was demonstrated to correlate to infarct size[17]. Accordingly, we found strong increases of serum troponin levels following LAD ligation. There was, however, no difference between control and lidocaine treated animals (Fig 3A). In line with these findings, histological infarct size was comparable in mice of the control and the lidocaine group (Fig 3B and 3C).

### Lidocaine Rescues Contractility of the Ischemic Region

**Fig 4:**
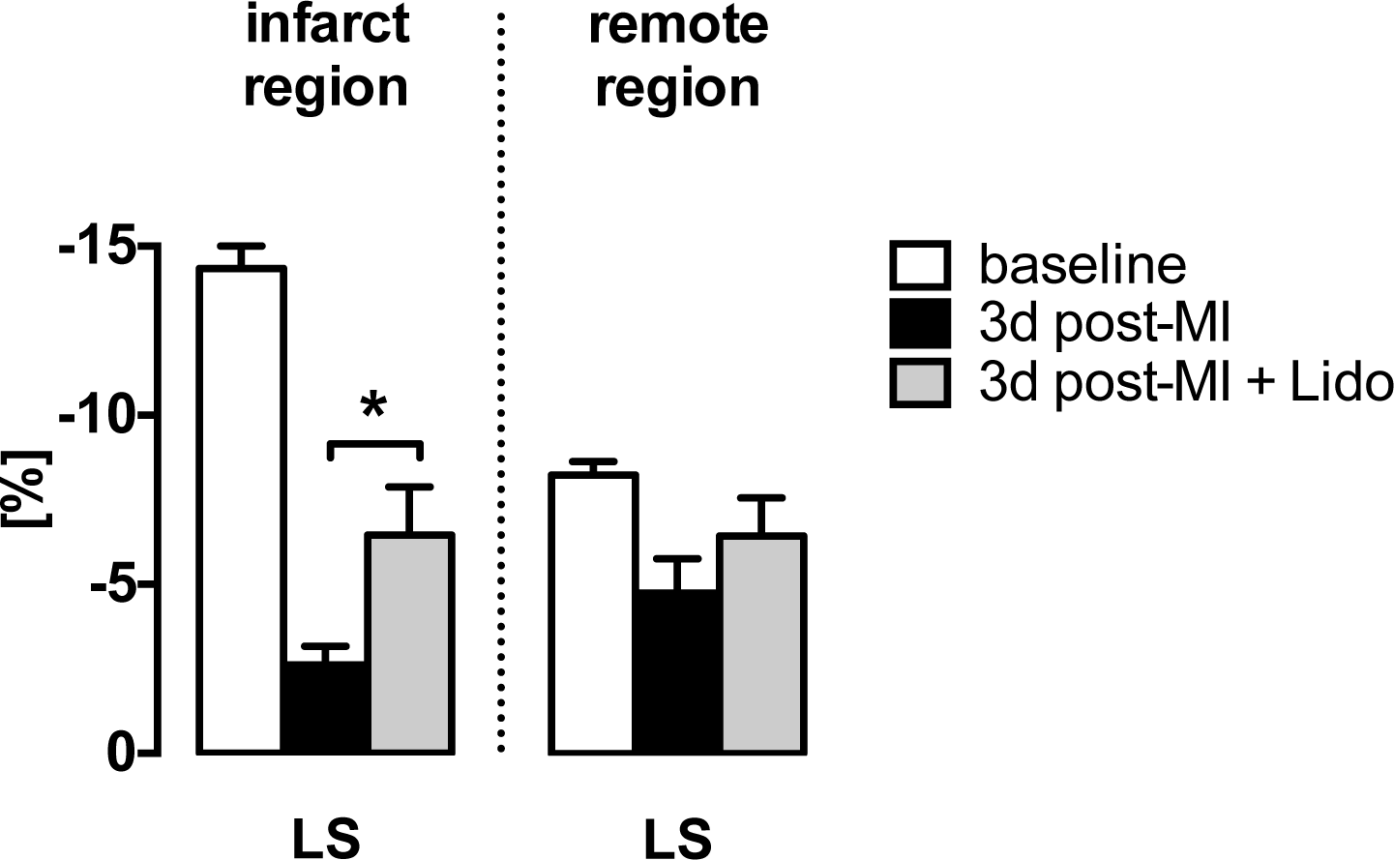
Lidocaine improves residual function of ischemic myocardium. Regional LS of segments of the infarcted region (MA, AA, AI) and the non-ischemic, remote region (BI, MI) are shown. Treatment with lidocaine results in improved LS following LAD ligation in the infarcted region (gray bars) as compared to mice receiving vehicle (black bars), while no effect of lidocaine is apparent in the remote region. *LS longitudinal strain*, *MA mid-anterior*, *AA apical-anterior*, *AI apical-inferior*, *BI basal-inferior*, *MI mid-inferior;* *p = 0.0167.

As infarct sizes did not differ between the control and lidocaine group, regional systolic function was investigated by speckle tracking. Only LV segments of the ischemic region demonstrated improved residual contractility with lidocaine treatment (p = 0.0167). In contrast, no effect of lidocaine was demonstrated in non-ischemic, viable regions (Fig 4). In line with these findings, there was no effect of lidocaine on global LV function in sham operated animals that were not subjected to myocardial ischemia (S5 Fig).

## Discussion

Perioperative myocardial ischemia remains a clinical challenge as an exceedingly high bleeding risk limits the use of pharmacological lysis therapy or interventional revascularisation in these patients. Targeting the intracellular sodium homeostasis during ischemia was reported to be beneficial in *ex vivo*- or short-term *in vivo*-models focusing on histological parameters of cardiac injury[7,8,18]. We here provide for the first time functional *in vivo* data, which demonstrate that blockade of voltage-gated sodium channels with lidocaine attenuates deterioration of global heart function by improving the residual contractility in ischemic regions of the heart.

Previous work on the effect of sodium channel blockade during myocardial ischemia was limited to *ex vivo* studies using isolated and perfused hearts[7,18], or to experiments, which addressed myocardial tissue damage or serum markers of cardiomyocyte cell death [8,19].

In our study, we addressed for the first time the functional impact of lidocaine treatment on cardiac function in an *in vivo* model of myocardial infarction. We took advantage of the most recent small-animal echocardiography techniques that allow detailed phenotyping of global and regional heart function, while limiting physiological alterations due to prolonged sedation, as needed for cardiac magnetic resonance imaging, to a minimum[15]. We found that LAD ligation leads to a vast decrease in conventional echocardiographic parameters of global systolic left ventricular function (EF and FAC) within 72h, which was even more pronounced using novel speckle tracking (LS). This confirms findings from Bauer et al., who also report a higher sensitivity for alterations of systolic myocardial function using speckle tracking[15].

Subcutaneous application of lidocaine twice daily rescued global systolic function in our model. The beneficial effect was evident in conventional echocardiographic measurements as well as in speckle tracking analysis of LS.

Significant systolic dysfunction is often associated with an impaired left ventricular filling during diastole. We found that LAD ligation leads to a strong decrease in late mitral inflow (A) and late mitral valve velocity (Aa) and consecutively increase in the E/A- and Ea/Aa ratios as signs of impaired diastolic function[20,21]. Treatment with lidocaine partially rescued diastolic function. While a clinical disease entity of diastolic heart failure with preserved systolic function as well as a respective animal models exists[20], diastolic dysfunction in our model occurs in conjunction with a sudden drop in systolic function. Our current data therefore does not differentiate whether the effect of VGSC blockade with lidocaine on diastolic function is merely a consequence of an improved systolic function in these mice or independent of this. However, inhibition of persistent sodium current had been demonstrated to decrease LV diastolic pressure [22–24], making it likely that the observed effects on diastolic function in our model are, at least partially, mediated by a direct effect of VGSC blockade with lidocaine.

Lidocaine had been suggested to reduce both infarct size and the release of troponins as serum markers of cardiomyocyte cell death in animal models of myocardial ischemia and reperfusion[8]. Kaczmarek et al. further observed that in the same experimental setup, application of lidocaine only during reperfusion was sufficient to reproduce the tissue-protective effect. The beneficial mechanisms of VGSC blockade with lidocaine that lead to a reduction of infarct size and troponin release during myocardial ischemia and reperfusion therefore seem to be most important during the phase of reperfusion. In line with these findings, we were unable to detect differences in either infarct size or troponins in our model of sustained myocardial ischemia.

Kaczmarek et al. further reported that reductions in infarct size and troponin release were due to a decrease in cardiomyocyte apoptosis in the lidocaine group. Previous findings demonstrated that cardiomyocyte cell death differs between ischemia and reperfusion: while cell death during ischemia is thought to be mainly due to necrosis[25], restoration of blood flow allows to fulfill cellular energy needs required for completion of apoptosis, hence accelerating cell death during reperfusion[26]. In our model, lidocaine did not alter cell death and infarct size which might be related to a predominant necrotic cell death due to sustained ischemia.

The mode of delivery of lidocaine might also contribute to the observed differences, as Kaczmarek et al used a continuous infusion during their short-term model as compared to subcutaneous injection in 12h-intervalls in the current study. While osmotic micropumps for use in mice that would allow a continuous release over several days are now available, simultaneous LAD ligation and implantation of such pump would significantly increase the burden on the animals and presumably mortality.

Volatile anesthetics such as isoflurane that was used in the current study are well known for their cardioprotective properties in tissue ischemia when applied before the ischemic event (preconditioning)[27]. We used isoflurane for anesthesia during LAD ligation, during postoperative echocardiography 72h after LAD ligation and during baseline echocardiography that was performed 48h before LAD ligation. To keep the influence of anesthesia as small as possible, isoflurane was strictly kept at 2% at all time points and in all study groups. However, we cannot exclude that the initial exposure to isoflurane during baseline echocardiography might have already reduced ischemic cardiac tissue damage resulting in no apparent effect on infarct size with further protection with lidocaine.

We observed differences in global systolic and diastolic function although infarct sizes were comparable in both, control and treatment groups. Analysis of regional wall motion of non-ischemic segments of the left ventricle by speckle tracking showed unaltered function with lidocaine treatment. However, segments of the ischemic region demonstrated improved residual contractility with lidocaine treatment, accounting for improved overall cardiac function. Blockade of VGSC was demonstrated before to decrease diastolic LV pressure *ex vivo* [22–24]. In line, we found preserved diastolic function in lidocaine-treated mice. Ischemic myocardium is particularly vulnerable to diastolic dysfunction, as the associated increase in wall tension compresses the vascular bed and reduces remaining coronary and myocardial blood flow during diastole. This effect might account for the observed improved contractility that is pronounced in the ischemic regions. Consistently, lidocaine did not affect cardiac function in the non-ischemic heart of sham-operated animals.

Voltage-gated sodium channels are involved in the rapid depolarisation during the phase 0 of the action potential and VGSC-blocking drugs can therefore affect impulse propagation. While class Ib antiarrhythmics such as lidocaine have in general only little proarrhythmic effects, we cannot exclude fatal arrhythmias in mice of our treatment group as continuous ECG-monitoring was not available in our animal facility.

In summary, we extend previous studies by demonstrating that blockade of VGSC with lidocaine improves global LV systolic and diastolic function in myocardial ischemia *in vivo* by sustaining residual contractility of the ischemic myocardium. Lidocaine treatment might therefore be beneficial as a bridging therapy to revascularisation in perioperative myocardial ischemia until revascularisation can be performed with a tolerable risk for bleeding.

## Supporting Information

S1 File Animal care and severity score sheet. (PDF)

S1 Fig Experimental protocol. (TIF)

S2 Fig Survival analysis. Kaplan-Meier curves are shown for postoperative survival in control (solid line) and lidocaine-treated mice (dashed line) during the observation period of 72h. The survival curves are compared using the log rank test (p = 0.77).
(TIF)

S3 Fig Echocardiographic validation of myocardial ischemia model. 12 to 16 weeks old BALB/c mice were subject to LAD ligation via left-sided thoracotomy. Echocardiography was performed before ligation (white bars) and 72h postoperative (black bars). **A** LAD ligation lead to a strong decrease in conventional echocardiographic parameters of EF (**p<0.0001) and FAC (**p<0.0001), as well as speckle-tracking measurement of LS (**p<0.0001). **B** Percentage change following LAD ligation was higher for speckle-tracking derived LS than for conventional measurement of of EF (= 0.0432), but not FAC (p>0.05). **C + D** Regional quantification of LS revealed the most pronounced change in segments of the infarcted region (mid-anterior [MA], apical-anterior [AA] and apical-inferior [AI]) as compared to remote, non-ischemic segments (basal-inferior [BI], mid-inferior [MI]) *p<0.05, **p<0.001 in C; **p = 0.0002 in D. *EF ejection fraction*, *FAC fractional area change*, *LS longitudinal strain*, *BA basal-anterior*, *MA mid-anterior*, *AA apical-anterior*, *AI apical-inferior*, *MI mid-inferior*, *BI basal-inferior*.
(TIF)

S4 Fig Diastolic function in myocardial ischemia. **A** Early (E) and late (A) mitral inflow velocites and E/A ratio are given. LAD ligation leads to a drop in late mitral inflow velocity (p<0.0001) and consecutive rise in E/A (**p = 0.0037). **B** Early (Ea) and late (Aa) mitral valve anulus velocities and Ea/Aa ratio are given. As shown for mitral inflow velocities, mitral valve anulus late velocity (Aa) drops following LAD ligation (p = 0.008) with a consecutive rise in Ea/Aa ratio (**p = 0.0023). White bars show preoperative values, black bars show values 3 days postoperative.
(TIF)

S5 Fig Lidocaine does not affect systolic function in non-ischemic hearts. Percentage changes to preoperative values 3 days following sham operation in animals without and with lidocaine treatment are shown. *EF ejection fraction; FAC fractional area change*
(TIF)
