# Supplementary material for: Lidocaine Enhances Contractile Function of Ischemic Myocardial Regions in Mouse Model of Sustained Myocardial Ischemia
Source: PLoS One. 2016 May 3;11(5):e0154699. doi: 10.1371/journal.pone.0154699 (PMC4854463; doi:10.1371/journal.pone.0154699)
Supplement: S1 File — (PDF) [file pone.0154699.s001.pdf]

Monitoring times:
Day 0 (= day of surgery): once after surgery
Day 1-3: 2x/day

Score Points (Description):
Activity
Normal activity =1
Retarded activity = 2
Sparse activity = 3
Almost no activity =4

Posture
Normal posture =1
Infrequent hunched posture = 2
Frequent hunched posture, labored breathing = 3
Almost always hunched posture=4

Mouse grimace Scale (MGS)
For scoring see below
->Total score (=activity + posture + MGS; eg. Minimal score=3, max. score=11):
- Total score = 6: analgetic treatment. If total score doesn't improve within 24 hours-> euthanize animal
- Total score = 7: analgetic treatment, control animal at least twice/day, inform surgeon. If total score doesn't improve within 12 hours-> euthanize animal
- Total score = 8: euthanize

Weight:
measure weight at day 0, 2, 3
weight loss > 10 % -> inform surgeon, weigh animal daily
weight loss > 15% or more plus one other clinical sign -> euthanize
weight loss >20% euthanize animal
Remarks:
as long as they fit in the line, write remarks right next to the respective animal
otherwise, remarks should be consecutively numbered (1,2,3,......) and
text should be written in the box at the bottom of the page

Mouse grimace Scale (MGS)

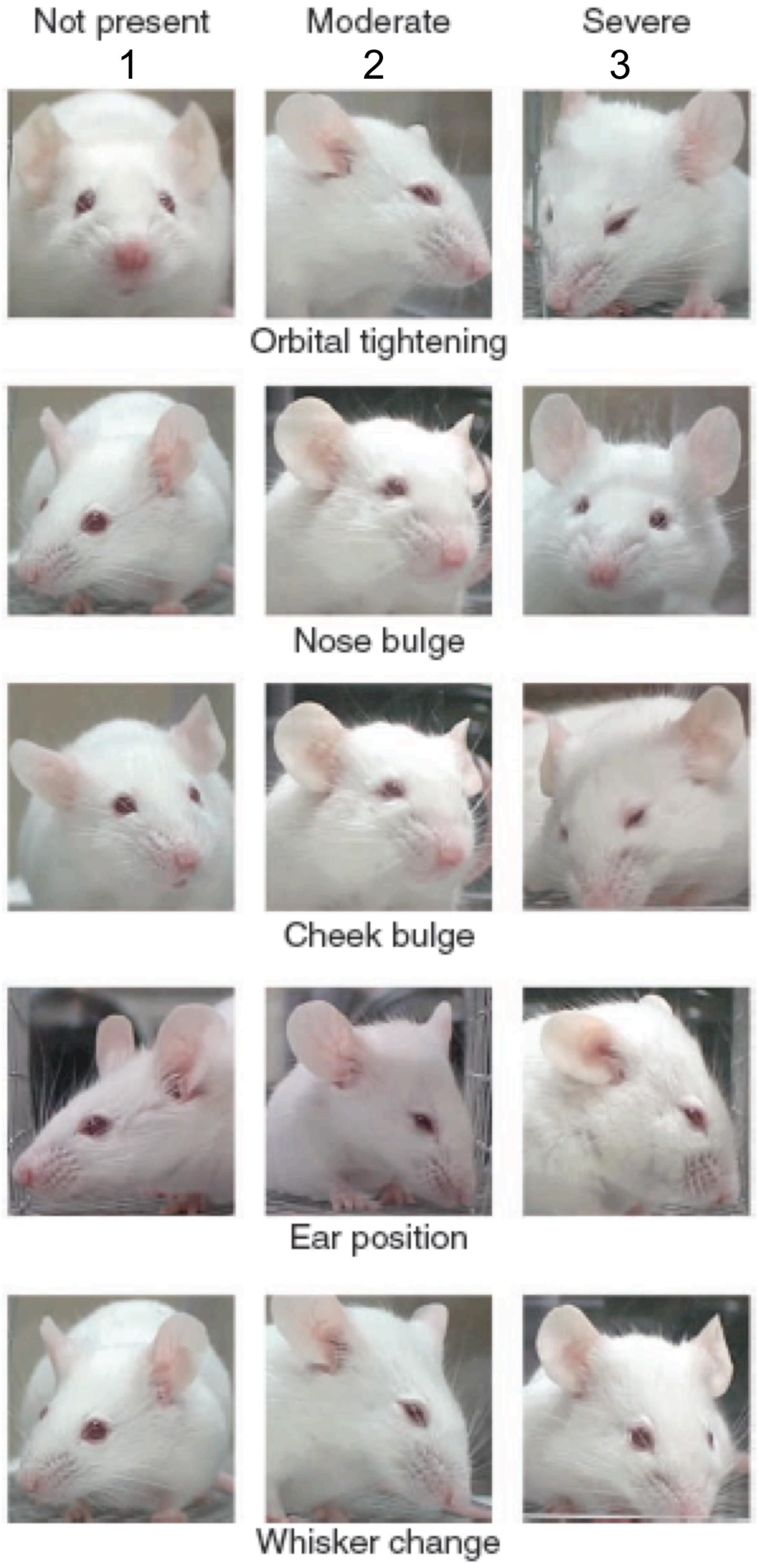

from: Nature Methods 7, 447 - 449 (2010)
