# Supplementary figures and images for: Lidocaine Enhances Contractile Function of Ischemic Myocardial Regions in Mouse Model of Sustained Myocardial Ischemia
Source: PLoS One. 2016 May 3;11(5):e0154699. doi: 10.1371/journal.pone.0154699 (PMC4854463; doi:10.1371/journal.pone.0154699)

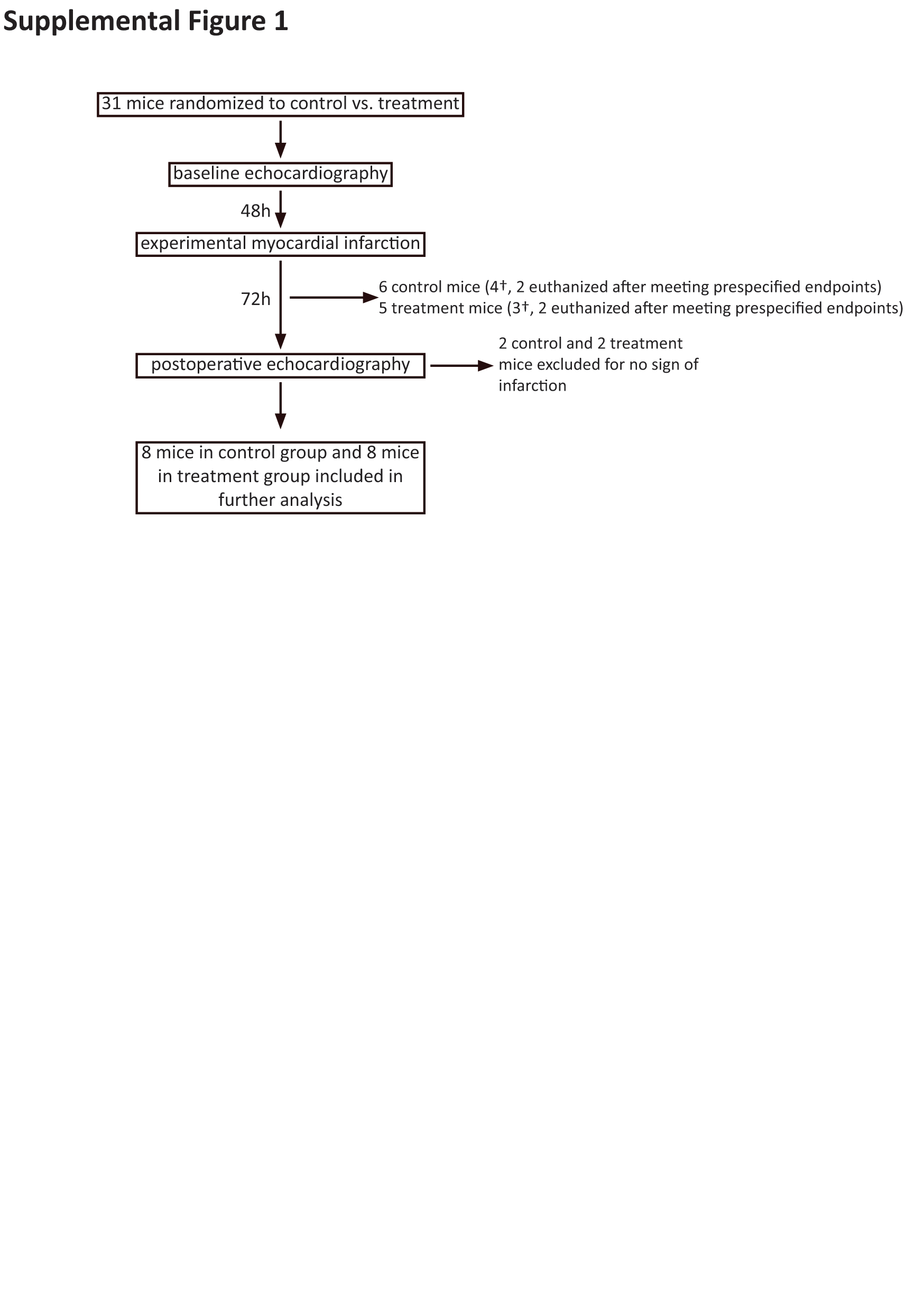

Supplement: S1 Fig — (TIF) [file pone.0154699.s002.tif]

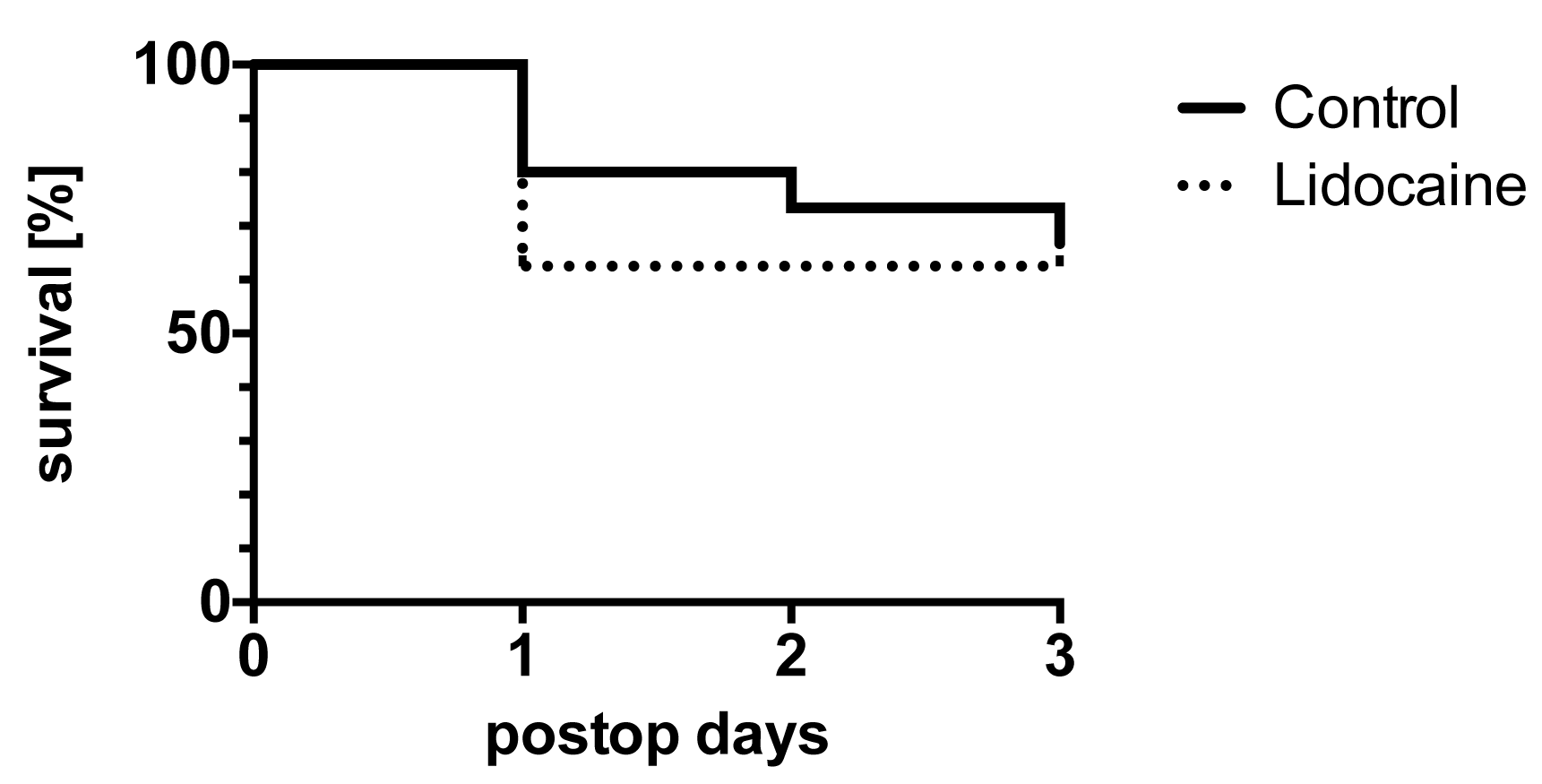

Supplement: S2 Fig — Kaplan-Meier curves are shown for postoperative survival in control (solid line) and lidocaine-treated mice (dashed line) during the observation period of 72h. The survival curves are compared using the log rank test (p = 0.77). (TIF) [file pone.0154699.s003.tif]

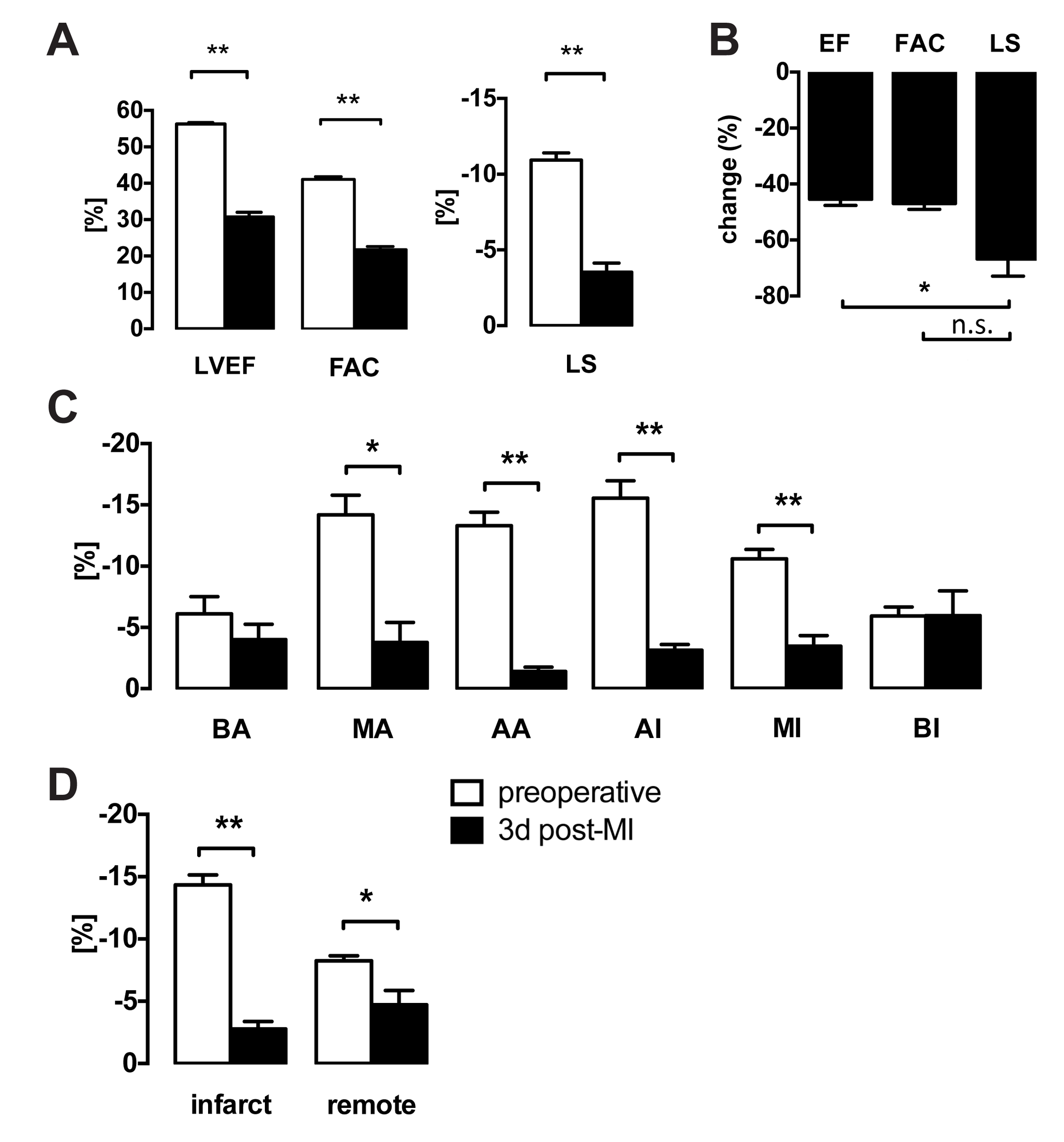

Supplement: S3 Fig — 12 to 16 weeks old BALB/c mice were subject to LAD ligation via left-sided thoracotomy. Echocardiography was performed before ligation (white bars) and 72h postoperative (black bars). A LAD ligation lead to a strong decrease in conventional echocardiographic parameters of EF (**p<0.0001) and FAC (**p<0.0001), as well as speckle-tracking measurement of LS (**p<0.0001). B Percentage change following LAD ligation was higher for speckle-tracking derived LS than for conventional measurement of of EF (= 0.0432), but not FAC (p>0.05). C + D Regional quantification of LS revealed the most pronounced change in segments of the infarcted region (mid-anterior [MA], apical-anterior [AA] and apical-inferior [AI]) as compared to remote, non-ischemic segments (basal-inferior [BI], mid-inferior [MI]) *p<0.05, **p<0.001 in C; **p = 0.0002 in D. EF ejection fraction, FAC fractional area change, LS longitudinal strain, BA basal-anterior, MA mid-anterior, AA apical-anterior, AI apical-inferior, MI mid-inferior, BI basal-inferior. (TIF) [file pone.0154699.s004.tif]

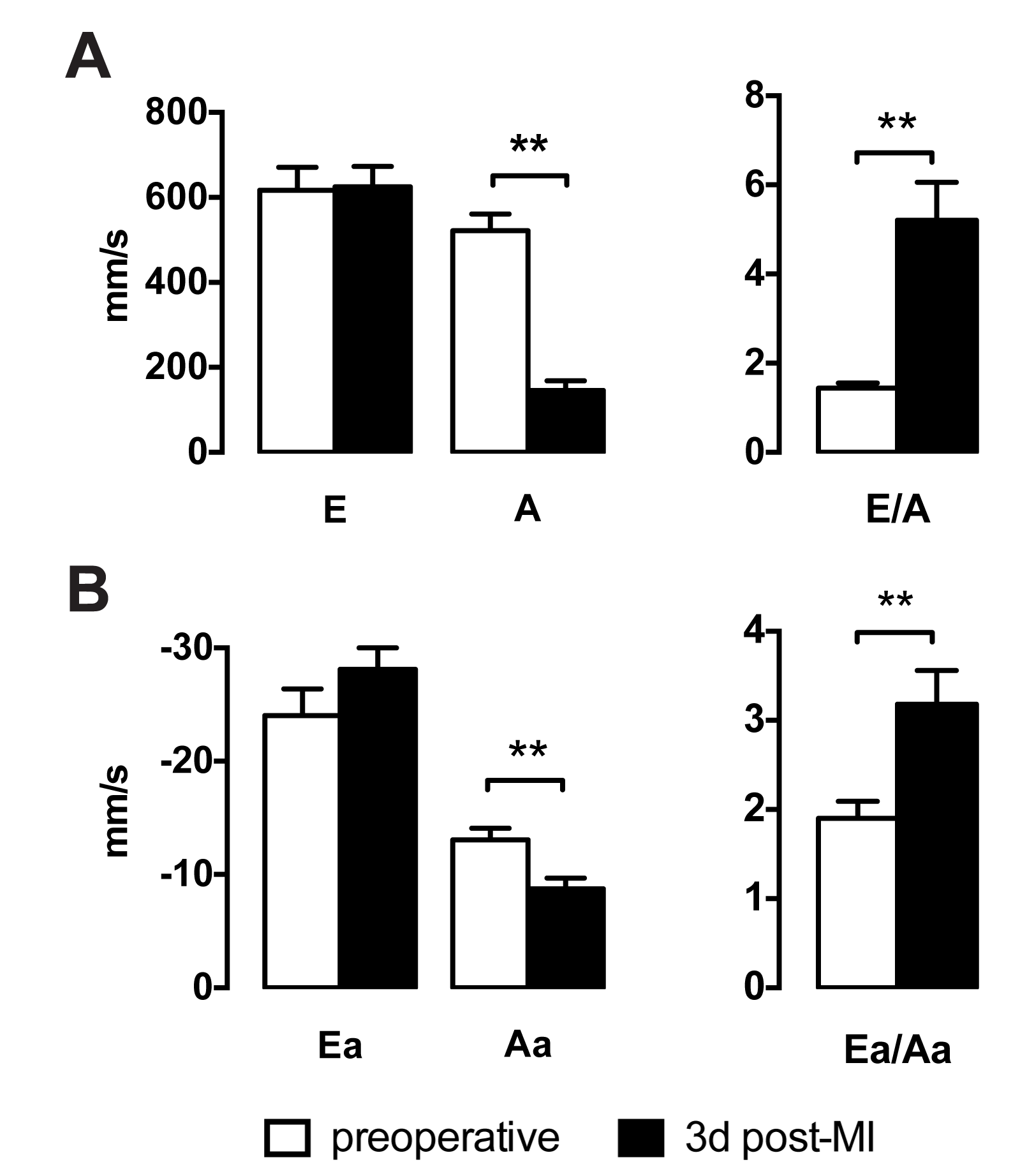

Supplement: S4 Fig — A Early (E) and late (A) mitral inflow velocites and E/A ratio are given. LAD ligation leads to a drop in late mitral inflow velocity (p<0.0001) and consecutive rise in E/A (**p = 0.0037). B Early (Ea) and late (Aa) mitral valve anulus velocities and Ea/Aa ratio are given. As shown for mitral inflow velocities, mitral valve anulus late velocity (Aa) drops following LAD ligation (p = 0.008) with a consecutive rise in Ea/Aa ratio (**p = 0.0023). White bars show preoperative values, black bars show values 3 days postoperative. (TIF) [file pone.0154699.s005.tif]

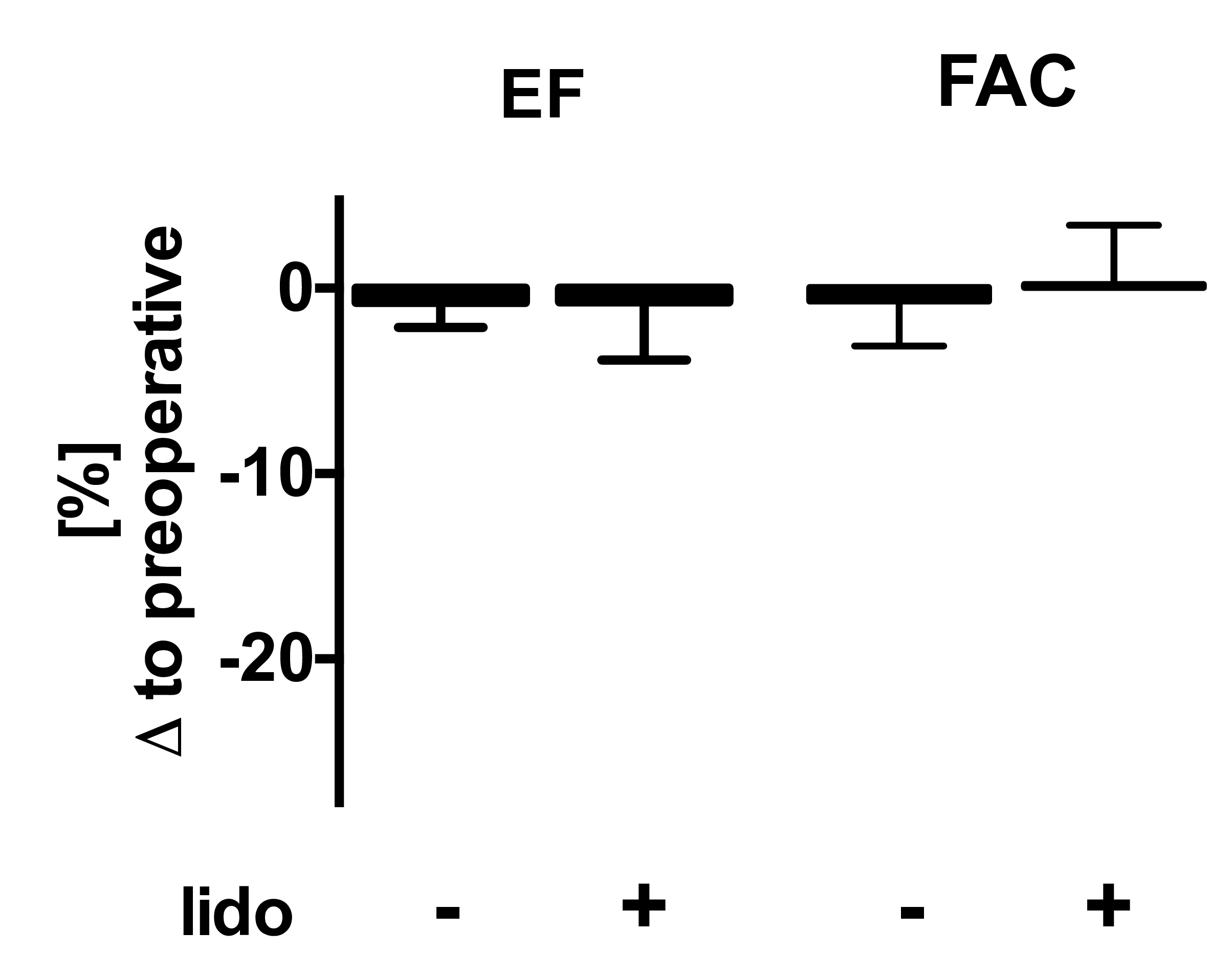

Supplement: S5 Fig — Percentage changes to preoperative values 3 days following sham operation in animals without and with lidocaine treatment are shown. EF ejection fraction; FAC fractional area change (TIF) [file pone.0154699.s006.tif]
